# Ion-concentration gradients induced by synaptic input increase the voltage depolarization in dendritic spines

**DOI:** 10.1007/s10827-024-00864-4

**Published:** 2024-02-13

**Authors:** Florian Eberhardt

**Affiliations:** 1 Faculty of Biology, Ludwig-Maximilians-Universität München, Großhaderner Straße 2, Planegg-Martinsried, 82152 Germany; 2 Bernstein Center for Computational Neuroscience, Großhaderner Straße 2, Planegg-Martinsried, 82152 Germany

**Keywords:** Dendritic spines, Cable theory, Electrodiffusion, Ion-concentrations, Diffusion currents, Spine neck resistance

## Abstract

The vast majority of excitatory synaptic connections occur on dendritic spines. Due to their extremely small volume and spatial segregation from the dendrite, even moderate synaptic currents can significantly alter ionic concentrations. This results in chemical potential gradients between the dendrite and the spine head, leading to measurable electrical currents. In modeling electric signals in spines, different formalisms were previously used. While the cable equation is fundamental for understanding the electrical potential along dendrites, it only considers electrical currents as a result of gradients in electrical potential. The Poisson-Nernst-Planck (PNP) equations offer a more accurate description for spines by incorporating both electrical and chemical potential. However, solving PNP equations is computationally complex. In this work, diffusion currents are incorporated into the cable equation, leveraging an analogy between chemical and electrical potential. For simulating electric signals based on this extension of the cable equation, a straightforward numerical solver is introduced. The study demonstrates that this set of equations can be accurately solved using an explicit finite difference scheme. Through numerical simulations, this study unveils a previously unrecognized mechanism involving diffusion currents that amplify electric signals in spines. This discovery holds crucial implications for both numerical simulations and experimental studies focused on spine neck resistance and calcium signaling in dendritic spines.

## Introduction

Synapses on dendritic spines are the major recipients of excitatory input to pyramidal neurons (Megıas et al., 2001). During activation of the synapse, synaptic currents depolarize the spine head (Acker et al., 2016). The synaptic currents are then transmitted to the parent dendrite, and the collective input to the neuron gets integrated along the dendritic tree (Branco & Häusser, 2011). However, electric signals can also be transmitted in the opposite direction. A back-propagating action potential (bAP) is generated at the axon hillock and travels up the dendritic tree and finally depolarizes the cell membrane in dendritic spines (Stuart et al., 1997). Additionally, dendritic spikes, namely dendritic sodium, calcium, or N-methyl-D-aspartate (NMDA) spikes exist. In contrast to somatic action potentials, these local regenerative potentials can be triggered in the dendritic tree (Larkum et al., 2022). While spines can compartmentalize electrical and chemical signals evoked by synaptic input (Bell et al., 2019; Cornejo et al., 2022; Higley & Sabatini, 2012), postsynaptic signals (bAPs or dendritic spikes) are assumed to invade the spines without attenuation (Lee et al., 2012; Yuste, 2013). The integration of signals related to pre- and postsynaptic activity is of great interest, as the activation of voltage-dependent NMDA receptors in dendritic spines can induce synaptic plasticity through calcium influx (Basu & Lamprecht, 2018; Nevian & Sakmann, 2006). To study electric signals propagating along a dendrite the cable equation can be used (Rall, 1995). The assumptions of cable theory are well-justified for dendrites, but unfortunately the formalism fails when applied to very small structures such as dendritic spines (Qian & Sejnowski, 1989).

The cable equation models the electric potential along a dendrite or an axon by its membrane capacitance, intracellular resistance and membrane resistivity. However, the ionic composition of the intracellular electrolyte is assumed to be constant and currents induced by the diffusion of ions are ignored. Due to the small and restricted volume of spines, their intracellular ion concentrations can easily change (Lagache et al., 2019). A typical pyramidal cell spine head, with a volume of $0.02~ \mu m^3$ (Eberhardt et al., 2022), is estimated to contain approximately $1.2\cdot 10^5$ sodium ions^1^ at a concentration of 10 mM. During an excitatory postsynaptic potential (EPSP) with a synaptic current of 23 pA (Cornejo et al., 2022) that lasts for 12 ms (Acker et al., 2016), the estimated number of sodium ions entering the spine^2^ is roughly $17.2 \cdot 10^5$. The number of sodium ions entering the spine head during a synaptic event is therefore significantly higher than the number of sodium ions located inside the spine head at rest.

The resulting concentration change in the spine head persists for several milliseconds, even in the case of small ions. The decay time $\tau$ for changes in sodium concentration in the spine head can be estimated (Tønnesen & Nägerl, 2016) based on the diffusion coefficient of sodium *D*, the head volume $V=\frac{4}{3}\pi R^3$, the length *L* of the neck, and the cross-sectional area $A=\pi R^2$ of the spine neck (where *R* is the radius) by $\tau = \frac{V \cdot L}{A \cdot D}$. For a typical pyramidal cell spine with a neck diameter of 50 nm, a neck length of 500 nm, a head radius of $r_{\text {Head}} = 250$ nm (Tønnesen et al., 2014; Arellano et al., 2007), and a diffusion constant of $D_{\text {Na}} = 0.5 \mu \text {m}^2/\text {ms}$ (Lagache et al., 2019; Samson et al., 2003), the decay time for sodium gets estimated to be 8 ms.

The concentration gradients will induce electric diffusion currents across the neck that are not captured by cable theory (Qian & Sejnowski, 1989). Therefore Poisson-Nernst-Planck (PNP) equations (MacGillivray, 1968) have been used to accurately model the electric function of dendritic spines (Boahen & Doyon, 2020). In modeling electric signals in spines, previous studies have shown that the 3D spine geometry can be simplified into a 1D cable without any problems. However, the resulting models are either still a combination of Poisson and Nernst-Planck equations, necessitating complex finite volume methods (Breit & Queisser, 2021), or they make strong simplifications to the composition of the electrolyte (Lagache et al., 2019). Especially in time dependent systems a numerical implementation of the PNP formalism remains non-trivial and computationally expensive. As a consequence, very often only small and extremely simplified systems are studied (Cartailler et al., 2017; Pabst, 2014).

Inspired by previous studies, it is shown here that an extension to cable theory, including diffusion currents, can be derived building on an analogy between the electric potential and the chemical potential. The resulting equations consider not only the electric potential but also the time-dependent concentrations of various ion species with individual diffusion constants. The presented system of equations resembles the Nernst-Planck equations (Cohen & Cooley, 1965) but no longer includes the Poisson equation. Very similar equations have successfully been used to study calcium signals in spines and dendrites (Bywalez et al., 2015; Li, 2023). The major advantage over the PNP-equations is that a simple numerical algorithm based on finite differences is sufficient to reliably find solutions even in systems with multiple ion species and at the lowest spatial resolution. The simulation results are consistent with previous experimental findings on electric signals in spines (Cornejo et al., 2022), and confirm results from other simulation studies that synaptic currents significantly alter ion concentrations in the spine head. Most importantly, this study uncovers a previously unnoticed mechanism central to boosting electric signals in spines. An imbalance in diffusion currents, arising from concentration gradients across the spine neck, influences drift currents, intensifying the depolarization of the spine head. This additional depolarization complements that induced by synaptic currents, significantly elevating the spine membrane voltage by tens of percentage points. These revelations hold significant implications for interpreting computer simulation results, investigating calcium influx into spines, and measuring spine neck resistance.

## Methods

The cable equation (Rall, 1977) describes how the temporal evolution of the electrical potential $\Phi$ along a passive neuronal process with length *l* and varying radius *a* depends on the membrane currents, Ohmic axial currents, and on the membrane capacitance. In this section it is demonstrated that these equation can be extended to include currents induced by the diffusion of intracellular ions. For the resulting equations a explicit algorithm based on finite differences is derived.

Similar conceptual derivations can be found in works such as (Henry et al., 2008), which derived fractional cable equations for dendrites. Previous studies have also employed similar equations to model calcium signals in dendrites (Li, 2023). Electrodiffusion models have been used to describe electric signals in spines (Qian & Sejnowski, 1989). However, the formalisms used in these studies still require complex numerical methods (Breit & Queisser, 2021) or make strong simplifications to the composition of the electrolyte (Lagache et al., 2019). In contrast, the equations and the finite difference scheme presented here allow for simple numerical solvers and accurate solutions independent of the composition of the electrolyte.

The subsequent derivation relies on an analogy between the electrical potential $\Phi$ and the chemical potential $\mu _k$ (where k denotes the ion species), incorporating the Nernst-Planck equation (Cohen & Cooley, 1965). The Nernst-Planck equation reads as

1 $$\vec{j_k} = -D_k (\vec{\nabla} n_k - \dfrac{n_k z_k e}{k_B T}\vec{\nabla}\Phi).$$

$\vec{j_k}$ describes the directed particle current density (number of particles per unit area per unit time) of an ion-species *k* with density $n_k$ (number of particles per unit volume), as a sum of diffusion induced by concentration gradients and drift of ions induced by the electric field $-\nabla \Phi$. $D_k$ denotes the diffusion constant, $z_k$ the charge number, *e* the elementary charge, $k_B$ the Boltzmann constant and *T* the temperature.

In the following a 1D-cable with radial symmetry and without radial dependence of the electrical field and the chemical potential gradient is considered. As indicated by Eq. (1), the total particle current $j_k$ of ion species *k* is a result of the diffusion current $j_{c,k}$ (electric current induced by the diffusion of ions) and the drift current $j_{e,k}$ (electric current induced by a gradient in the electric potential). To transform a particle current into an electrical current, one can simply multiply the particle currents $j_k$ by $z_k e$. The axial drift current (charge per unit time induced by the electric field) of an ion species *k* along a cylinder with a cross-section $A=\pi a^2$ (and radius *a*) is therefore:

2 $$\begin{aligned} j_{e,k} \pi a^2 e z_k = -\dfrac{D_k e^2 z_k^2 \pi a^2}{k_B T}n_k\dfrac{\partial \Phi }{\partial x}. \end{aligned}$$

Now define

$$\begin{aligned} I_e =\sum _k j_{e,k} \pi a^2 e z_k \end{aligned}$$

as the total axial electric drift current (charge per unit time induced by the electric field) and

3 $$\begin{aligned} r_e=\dfrac{k_B T}{\sum _k D_k e^2 z_k^2 n_k} \end{aligned}$$

as the electrical resistivity for electric drift currents (unit ohm metre). Together with Eq. (2) this leads to

4 $$\begin{aligned} I_e = - \dfrac{\pi a^2}{r_e} \dfrac{\partial \Phi }{\partial x}. \end{aligned}$$

The chemical potential $\mu _k$ of an ion-species *k* if given by

5 $$\begin{aligned} \mu _k = k_B T \ln \left( \dfrac{n_k}{n_{ref}} \right) . \end{aligned}$$

$n_{ref}$ is a reference concentration. The gradient of the chemical potential is simply

6 $$\begin{aligned} \dfrac{\partial \mu _k}{\partial x} = \dfrac{k_B T}{n_k}\dfrac{\partial n_k}{\partial x}. \end{aligned}$$

According to the Nernst-Planck equation, the axial electric diffusion current (charge flux per unit time) induced by the diffusion of ion species *k* can be computed as

7 $$\begin{aligned} I_{c,k} = - j_{c,k} \pi a^2 e z_k = - D_k e z_k \pi a^2 \dfrac{\partial n_k}{\partial x}, \end{aligned}$$

where $j_{c,k}$ is the charge flux of ion species k per unit area per unit time, induced by diffusion.

Then one defines the diffusional resistivity for electric diffusion currents (unit ohm metre coulomb)

8 $$\begin{aligned} r_{c,k} = r_{e,k}e z_k = \dfrac{k_B T}{D_k e z_k n_k}, \end{aligned}$$

in analogy to the electrical resistance $r_{e,k}$ and insert it into Eq. (7). Together with Eq. (6) this leads to

9 $$\begin{aligned} I_{c,k} = - \dfrac{\pi a^2}{r_{c,k}} \dfrac{\partial \mu _k}{\partial x}. \end{aligned}$$

**Fig. 1:**
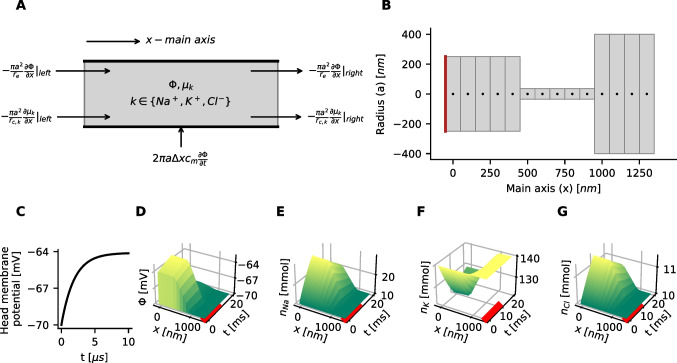
Simulation of an idealized dendritic spine based on parameters found in (Cornejo et al., 2022) **A** In cable theory the axial electric currents are computed from the gradient of the electric potential along the cable. By an analogy between the electric potential $\phi$ and the chemical potential $\mu$ the same formalism can be developed for the diffusion of ions. **B** The solution is computed on a lattice with a finite-difference algorithm. The one-dimensional spatial lattice is indicated by the black dots. Neumann boundary conditions are used to inject a sodium current to the spine head on the left side (red line) and to establish no-flux boundaries for potassium and chloride, and the electrical potential. On the right dendritic end, the ion concentrations are set to constant values. **C**-**E** The membrane potential and the concentrations of sodium, potassium and chloride is simulated on the spatial lattice with forward time stepping. A 25 *pA* current of sodium ions gets injected for 10 *ms* (red bar). **C** The time course of the membrane potential shows a rapid charging of the membrane capacitor within a few microseconds, measured in the leftmost segment of the spine head. **D** The injected current depolarizes the spine head by several millivolts. The voltage $\Phi$ drops almost completely across the spine neck. After the fast charging of the capacitor, the membrane voltage continues to slowly further increase. During the current injection, sodium $n_{Na}$ **E** and chloride $n_{Cl}$ **F** concentrations are increasing while the potassium $n_{K}$ **G** concentrations is decreasing in the simulated spine

Next, following the derivation of the cable equation, as described in (Dayan & Abbott, 2001), on subdivides the cable along the main axis into N discrete segments of identical size $\Delta x$. $x_i$ denote the central points of the segments (Fig. 1B). The electric potential $\Phi$ along the cable results from a very slight imbalance of positive and negative ions, charging the membrane. The electric potential $\Phi (x_i)$ of the cable segments at location $x_i$ can be computed based on the membrane capacitance

10 $$\begin{aligned} 2 \pi a \Delta x c_m \Phi = \pi a^2 \Delta x \sum _k z_k e n_k, \end{aligned}$$

Here, $c_m$ represents the specific capacitance of the membrane (capacitance per unit area). To ensure exact electro-neutrality at 0 mV membrane potential, a fixed constant negative background charge with density $n_0$ is incorporated into the model.

To derive the cable equation, one balances the capacitive current and all currents that enter (or leave) a cable segment of finite length $\Delta x$ (Fig. 1A). For simplicity, a system without any membrane currents is assumed here.

11 $$\begin{aligned} 2 \pi a \Delta x c_m \dfrac{\partial \Phi }{\partial t} =& - \left( \dfrac{\pi a^2}{r_e} \dfrac{\partial \Phi }{\partial x}\right) |_{left } + \left( \dfrac{\pi a^2}{r_e} \dfrac{\partial \Phi }{\partial x}\right) |_{right} \\& +\sum _k\left[ - \left( \dfrac{\pi a^2}{r_{c,k}} \dfrac{\partial \mu _k}{\partial x}\right) |_{left } + \left( \dfrac{\pi a^2}{r_{c,k}} \dfrac{\partial \mu _k}{\partial x}\right) |_{right} \right] \end{aligned}$$

This equation is divided by $2 \pi a \Delta x$ and the limit $\Delta x \rightarrow 0$ is taken. Further, the gradient of the chemical potential is replaced, using Eq. (6). Compared to its standard form (Dayan & Abbott, 2001), this leads to a cable equation with an additional diffusion term.

12 $$\begin{aligned} c_m \dfrac{\partial \Phi }{\partial t} = \dfrac{1}{2a} \dfrac{\partial }{\partial x} \left( \dfrac{a^2}{r_e} \dfrac{\partial \Phi }{\partial x} \right) +\sum _k\left[ \dfrac{1}{2a} \dfrac{\partial }{\partial x} \left( a^2 D_k e z_k \dfrac{\partial n_k}{\partial x} \right) \right] \end{aligned}$$

The same derivation can be carried out for the chemical potential to describe temporal changes in the ionic concentrations along the cable. First one sums over all particle currents entering or leaving the volume (Fig. 1A):

13 $$\begin{aligned} \pi a^2 \Delta x \dfrac{\partial n_k}{\partial t} =& - \left( \dfrac{\pi a^2}{r_{e,k}z_k e} \dfrac{\partial \Phi }{\partial x}\right) |_{left } + \left( \dfrac{\pi a^2}{r_{e,k}z_k e} \dfrac{\partial \Phi }{\partial x}\right) |_{right}\\& - \left( \dfrac{\pi a^2}{r_{c,k} z_k e} \dfrac{\partial \mu _k}{\partial x}\right) |_{left } + \left( \dfrac{\pi a^2}{r_{c,k} z_k e} \dfrac{\partial \mu _k}{\partial x}\right) |_{right} \end{aligned}$$

Then the above equation is divided by $\pi a^2 \Delta x$ and the limit $\Delta x \rightarrow 0$ is taken again, to arrive at

14 $$\begin{aligned} \dfrac{\partial n_k}{\partial t} = \dfrac{1}{a^2 z_k e} \dfrac{\partial }{\partial x} \left( \dfrac{a^2}{r_{e,k}} \dfrac{\partial \Phi }{\partial x} \right) + \dfrac{1}{a^2} \dfrac{\partial }{\partial x} \left( a^2 D_k \dfrac{\partial n_k}{\partial x} \right) . \end{aligned}$$

Equations (12) and (14), together with adequate boundary conditions and initial conditions, uniquely determine the solution. By setting $\dfrac{\partial n_k}{\partial t}=0$ one recovers the well known standard form of the cable equation.

### Numerical implementation

For K different ion-species, excluding the constant background charge, e.g. K=3 for $Na^+$, $K^+$ and $Cl^-$, the system of equations derived above (14), together with (12), consist of $K + 1$ equations. Considering, that $\Phi$ is a function of $n_k$ (Eq. (10)), and inserting its spatial derivative

15 $$\begin{aligned} \dfrac{\partial \Phi }{\partial x} = \dfrac{\partial }{\partial x} \sum _k \dfrac{a z_k e n_k}{2 c_m}. \end{aligned}$$

into (14), one has to solve a system of only *K* equations, effectively.

For numerical treatment, an explicit algorithm based on finite differences can be employed to solve this system of equations. To derive the update rule, one rewrites Eq. (14) with coefficients α and σ as

16 $$\begin{aligned} \dfrac{\partial n_k}{\partial t} = \alpha _{e,k} \dfrac{\partial }{\partial x} \left( \sigma _{e,k} \dfrac{\partial }{\partial x} v_e \right) + \alpha _{d} \dfrac{\partial }{\partial x} \left( \sigma _{d,k} \dfrac{\partial }{\partial x} v_{d,k} \right) . \end{aligned}$$

The two terms $\dfrac{\partial }{\partial x} \left( \sigma \dfrac{\partial }{\partial x} v \right)$ are then approximated based on a central difference scheme with $\Delta x = h$. After approximating the inner derivative $\left( \sigma \frac{\partial }{\partial x} v \right)$, one arrives at

17 $$\begin{aligned} \dfrac{\partial }{\partial x} \left( \sigma \dfrac{\partial }{\partial x} v \right) \approx \dfrac{\partial }{\partial x}\left( \dfrac{\sigma (x_i+h/2)v(x_i+h/2)-\sigma (x_i-h/2)v(x_i-h/2)}{h}\right) \end{aligned}$$

The positions $x_i$ are indicated by the black dots in Fig. 1B and represent the central points in each segment. *h* is the length of a segment and the distance between two consecutive points. The interfaces between two segments *i* and $j=i+1$ are located at positions $x_i+h/2$.

In the above equation, $\sigma (x_i+h/2)$ can be considered as the conductivity of the interface, that connects the segments *i* and $i+1$. The conductivity values of the interfaces are the average conductivity between $x_i$ and $x_i+h$, and can be computed as the harmonic mean of the conductivities of the connected segments *i* and $i+1$

18 $$\begin{aligned} \sigma (x_i+h/2) = \dfrac{\sigma (x_i)\sigma (x_i+1)}{\sigma (x_i)+\sigma (x_i+1)}. \end{aligned}$$

As $\sigma (x_i+h/2)$ is the value on the interface, it can be assumed that $\sigma (x_i+h/2)$ is constant between $x_i$ and $x_i+h$, and one can approximate the outer derivative based on a central difference scheme:

19 $$\begin{aligned} \dfrac{\partial }{\partial x} \left( \sigma \dfrac{\partial }{\partial x} v \right) &\approx \sigma (x_i+h/2)\dfrac{v(x_i+h)-v(x_i)}{h^2} \\&- \sigma (x_i-h/2)\dfrac{v(x_i)-v(x_i-h)}{h^2} \end{aligned}$$

Next, the left side of Eq. (14) can be approximated based on a forward difference scheme:

20 $$\begin{aligned} \dfrac{\partial n_k(x, t)}{\partial t} = \dfrac{n_k(x,t+\Delta t)-n_k(x,t)}{\Delta t} \end{aligned}$$

Combining the finite difference approximations of the left side and the right side of Eq. (14) leads to an update rule in which $n_k$ can be stepped forward in time:

21 $$\begin{aligned} n_k(x_i,t_k+\Delta t) = n_k(x_i,t_k) + \Delta t \left( A(x_{i-1},x_{i},x_{i+1},t_k) + B(x_{i-1},x_{i},x_{i+1},t_k) \right) \end{aligned}$$

Here *A* and *B* are

$$\begin{aligned} A(x_{i-1},x_{i},x_{i+1},t_k)=&\; \alpha _{e,k}(x_i) \sigma _{e,k}(x_i+h/2)\dfrac{v_e(x_i+h)-v_e(x_i)}{h^2}\\& - \sigma _{e,k}(x_i-h/2)\dfrac{v_e(x_i)-v_e(x_i-h)}{h^2} \end{aligned}$$

and

$$\begin{aligned} B(x_{i-1},x_{i},x_{i+1},t_k) .=&\; \alpha _{d}(x_i) \sigma _{d,k}(x_i+h/2)\dfrac{v_d(x_i+h)-v_d(x_i)}{h^2} \\&- \sigma _{d,k}(x_i-h/2)\dfrac{v_d(x_i)-v_d(x_i-h)}{h^2}, \end{aligned}$$

where $\alpha _{e,k}=\dfrac{1}{a^2 z_k e}$, $\alpha _d=\dfrac{1}{a^2}$, $\sigma _{e,k}=\dfrac{a^2}{r_{e,k}}$, $\sigma _{d,k}=a^2 D_k$, $v_e=\Phi$ and $v_{d,k}=n_k$. Code for numerical implementation is available at: https://github.com/feblmu/Eberhardt2024Ion-concentration.

### Boundary conditions and initial conditions

The spatial grid of the computational domain is shown in Fig. 1B. The region with a larger radius *a* on the right side represents the dendritic end, and on the left side, the synaptic end. The equispaced black dots are the central points $x_1$ to $x_N$ of the individual cylindrical segments. Each segment contains values for the electrical potential $\Phi (x_i, t)$ and the ion concentrations $n_k(x_i, t)$.

To apply the boundary conditions, one additional segment is appended on the left and right sides (not shown in Fig. 1B). The respective locations are $x_0$ and $x_{N+1}$.

Dirichlet boundary conditions are applied on the dendritic end, where the concentrations $n_k$ and the electric potential $\Phi$ at position $x=x_{N+1}$ are held constant. The ion concentrations are always held at their initial resting values $n_k^{rest}$. The electric potential $\Phi$ is set to the resting value $\Phi ^{rest}$ during most simulations. In simulations where the dendritic end gets depolarized, the value is set to a different value $\Phi (x_{N+1})=\Phi ^{depol}$.

Neumann boundary conditions are applied on the synaptic end (left side). To prevent flux of potassium and chloride through the synaptic end, the derivatives of the electric potential and the concentrations of chloride and potassium are set to zero at the synaptic end: $\frac{\partial \Phi }{\partial x} = 0$, $\frac{\partial n_{K}}{\partial x}=0$ and $\frac{\partial n_{Cl}}{\partial x}=0$. The boundary condition $\frac{\partial n_{Na}}{\partial x}=\frac{-I_{in}}{ (D_{Na} ez_{Na} \pi a^2)}$ forces the influx of sodium. Applied to the finite difference scheme, sodium influx can be realized by setting

$$\begin{aligned} n_{Na}(x=x_0,t)=n_{Na}(x=x_1,t)+h \frac{I_{in}}{ (D_{Na} ez_{Na} \pi a^2)}. \end{aligned}$$

Here, $I_{in}$ denotes the input current on the synaptic end of the computational domain (the red line in Fig. 1B), and *h* represents the spatial discretization, i.e., the distance between the central points of the segments.

As initial conditions at time $t=0$, the variables were set to the resting values $\Phi (x,t=0)=\Phi ^{rest}$ and $n_k(x,t=0)=n_k^{rest}$.

### Stability analysis

**Fig. 2:**
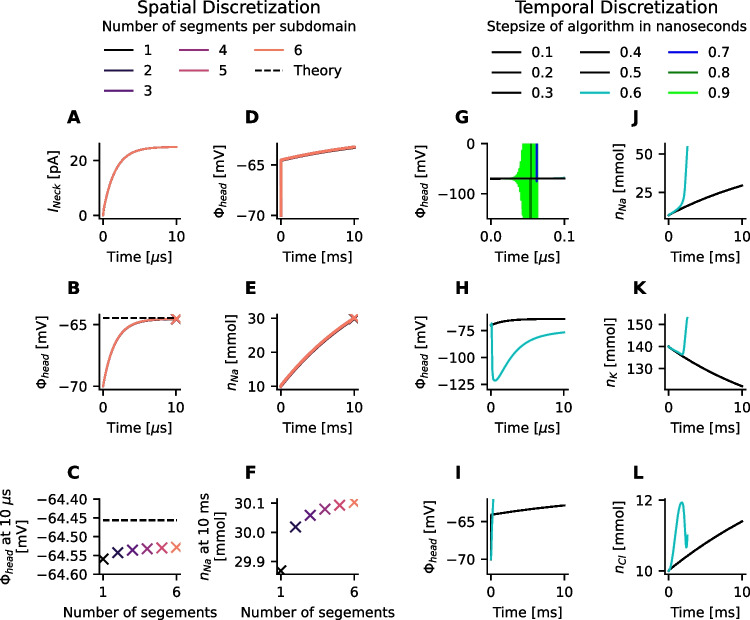
Analysis of accuracy and stability of the numerical solution. **A**-**F** A spine with a fixed length of 1.4 $\mu$m was discretized into a different number of cylindrical segments. The spine head, the spine neck, and the dendritic domain have the same length and contain the identical number of segments. A current of 25 pA gets injected for 10 ms. **A**, **B** The time course of the observed neck current $I_{\text {neck}}$ and spine head depolarization $\Phi _{\text {head}}$ (measured in the first segment of the head domain) are independent of spatial discretization during the phase of capacitor charging (lines are overlapping). **C** In all cases, a membrane potential that is close to the theoretically predicted value based on Ohm’s law is reached after 10 $\mu$s. **D**, **E** During 10 ms of synaptic current injection, the increase in membrane potential and the build-up of concentration changes measured in the first segment of the spine head are independent of spatial discretization (lines are overlapping). **F** The values of sodium concentrations $n_{\text {Na}}$ after 10 ms differ only very weakly. **G**-**L** The finite difference scheme was applied to a spine with 15 segments (5 segments per domain) while varying the size of the time steps. Again, a current of 25 pA gets injected for 10 ms. The stability of the solution depends on the time step, and solutions are stable if the time step is 0.5 ns or lower. Further decreasing the time step has no visible effect on the accuracy of the solution, as seen in the electric potential $\Phi _{\text {head}}$ and ion concentrations of sodium $n_{\text {Na}}$, potassium $n_K$, and chloride $n_{\text {Cl}}$. During the rest of this study, a spine with a total length of 1.4 $\mu$m was discretized into 5 head, 5 neck, and 4 dendritic segments of equal length. During all simulations, a time step of 0.1 ns was used. Only the radii of the cylindrical segments in the spine head and the spine neck were varied

A related finite difference scheme for a linear problem, the parabolic heat equation, indicates that the solution can achieve stability at low spatial and high temporal resolution (Landau et al., 2008). To analyze the accuracy and stability of the presented finite difference scheme, the spatial and temporal resolutions is varied (Fig. 2A-F). As stability is guaranteed by choosing a sufficiently low temporal resolution, the required temporal resolution for a fixed spatial discretization is determined by reducing temporal resolution until a stable solution is found (Fig. 2G-L).

### Simulation parameters

The simulation parameters were chosen as follows:

- Specific membrane capacitance: $c_m = 0.01~F/m^2$
- Temperature: $$T=310~K$$
- The resting potential was set to $\Phi ^{rest}=-70~mV$ For the analysis of the voltage-dependent NMDAR-channel current, $\Phi ^{rest}$ was set to $-85$ mV.
- Intracellular ion concentrations at rest: $n_{Na}^{rest}=140~mM$, $n_{K}^{rest}=10~mM$, and $n_{Cl}^{rest}=10~mM$
- The concentration of the fixed negative background charge $n_0$ was chosen according to Eq. (10) to ensure a resting potential $\Phi ^{rest}$ of $-70~mV$ (Milo et al., 2010).
- Diffusion constants: $D_{Na+}=0.65\cdot 10^{-9}\dfrac{m^2}{s}$, $D_{K+}=1.00\cdot 10^{-9}\dfrac{m^2}{s}$, and $D_{Cl-}=1.00\cdot 10^{-9}\dfrac{m^2}{s}$. Diffusion values are chosen in agreement with (Samson et al., 2003) and (Eberhardt, 2023).
- A spine with a total length of 1.4 $\mu$m was discretized into 5 head, 5 neck, and 4 dendritic segments of equal length.
- In all simulations, a time step of 0.1 ns was used.
- The radii of the cylindrical segments in the spine head and the spine neck were varied.

### Analysis

To analyze changes in electrical resistance, the total resistance of the spine for drift currents is computed by summing over all finite segments along the spine:

22 $$\begin{aligned} R_e =\sum _i\dfrac{r_e(x_i) \Delta x}{ A(x_i) } , \end{aligned}$$

where $r_e(x_i)$ denotes the electrical resistivity for drift currents in the *i*-th segment, and $A=\pi a^2$ represents the cross-sectional area.

Estimations of the spine neck resistance ($R_n$) in experiments often rely on a voltage divider model (Cornejo et al., 2022) given by:

23 $$\begin{aligned} R_n = \frac{V_{sp} - V_{den}}{I_{syn}}, \end{aligned}$$

where $V_{sp}$ is the experimentally measured membrane potential of the spine head. In the simulations conducted in this study, $V_{sp}$ is determined by the electric potential in the first segment of the spine head, denoted as $\Phi (x_1)$. Meanwhile, $V_{den}$ corresponds to the measured membrane potential in the dendritic shaft, estimated here as $\Phi (x_N)$. The parameter $I_{syn}$ represents the experimentally estimated synaptic current, and its value is controlled by the strength of the injected current ($I_{in}$) during the simulations.

To analyze NMDAR-channel currents, the model and all parameters of NMDAR-channel kinetics, but also the resting potential, are based on (Chiu & Carter, 2022). The other spine parameters are identical to those of the simulation shown in Fig. 1. The normalized channel conductance is

$$\begin{aligned} g_{NMDA}(V) = \dfrac{1}{1 + a \exp (b\cdot V)}, \end{aligned}$$

where *V* is the voltage difference across the synapse $\Phi (x_1)-\Phi (x_0)$. The normalized NMDAR-current gets approximated by Ohm’s law.

$$\begin{aligned} I_{NMDA} = g_{NMDA}(V) (\Phi (x_1,t)-\Phi ^{reversal}). \end{aligned}$$

The reversal potential ($\Phi ^{reversal}$) was set to 0 mV, and the parameters *a* and *b* were selected as $a=0.073$ and $b=-0.074.$  

## Results

Because of the small size of dendritic spines, even moderate ion influxes are likely to have a strong influence on the spatio-temporal dynamics of the ionic concentrations ($n_k$) within spines. Through the diffusion of ions, the concentration gradients will induce additional electrical currents. A system of coupled partial differential equations allows one to calculate the temporal evolution of the concentration of the three major ion species, i.e., sodium, potassium and chloride, along a cable.

24 $$\begin{aligned} \dfrac{\partial n_k}{\partial t} =&\; \dfrac{1}{a^2 z_k e} \dfrac{\partial }{\partial x} \left( \dfrac{a^2}{r_{e,k}} \dfrac{\partial \Phi }{\partial x} \right) \\&+ \dfrac{1}{a^2} \dfrac{\partial }{\partial x} \left( a^2 D_k \dfrac{\partial n_k}{\partial x} \right) ,~~\text {with}~~ r_{e,k}=\dfrac{k_B T}{D_k e^2 z_k^2 n_k} \end{aligned}$$

Here, $r_{e,k}$ is the electrical resistivity of ion-species *k*. $\Phi$ denotes the electric potential, *x* the position along the cable, *a* the radius of the cable, $D_k$ the diffusion constant, $z_k$ the charge number, *e* the elementary charge, $k_B$ the Boltzmann constant and *T* the temperature.

The electric potential can be computed from the summed charge of the ions sodium, potassium and chloride, a constant negative background charge and the specific membrane capacitance $c_m$:

25 $$\begin{aligned} \Phi = \dfrac{a}{2 c_m} \sum _k z_k e n_k \end{aligned}$$

### Ion concentrations in the spine head change during current injection

Recently, voltage compartmentalization in dendritic spines was measured *in vivo* using genetically encoded voltage indicators (Cornejo et al., 2022). Spines on pyramidal neurons in mouse somatosensory cortex were found to compartmentalize the membrane voltage at more than 5 mV on average. At an average photostimulation current of 23 pA, the neck resistance was estimated to be approximately $230~M\Omega$. The first simulation parameters are chosen accordingly, to reproduce these results. The spine neck diameter is set to 70 *nm* and a neck length to 500 *nm*. The neck resistance is estimated from the ion concentrations at rest to be $230~M\Omega$, using Eq. (3). The head volume is set to $0.01~\mu m^3$, to match the typical volume of the intracellular space of spines from hippocampal pyramidal cells, excluding organelles and cytoskeleton (Eberhardt et al., 2022). Then a constant influx of sodium ions that leads to an electric current of 25*pA* for a time of 10*ms* is injected. The duration of the current injection is chosen in agreement with the average time of synaptic opening of 12*ms* in basal dendrites of L5 pyramidal neurons in acute brain slices (Acker et al., 2016).

The simulations show that the membrane capacitor charges rapidly in just a few microseconds (Fig. 1C). This agrees with (Lagache et al., 2019). The near instantaneous charging of the membrane is also consistent with the extremely small membrane capacitance of the spine head. During rapid charging, the membrane voltage of the head converges to a depolarization of around 6 *mV*. Subsequently, over a longer timescale, the head’s depolarization increases to 7.2 *mV* during the full 10 *ms* of current injection. The electrical potential is almost constant along the spatial axis within the head and drops almost linearly along the neck (Fig. 1D). The ion concentrations undergo significant changes during current input. After 10 *ms* the sodium concentration increases from 10.0 *mM* to 28.6 *mM*, chloride from 10.0 *mM* to 11.4 *mM* and the potassium concentration drops from 140.0 *mM* to 122.0 *mM*. When the current injection ends, the electric potential drops within a few microseconds to a value of $-68.8~mV$, close to the resting voltage of 70 *mV*, as the membrane capacitor discharges. Then the electric potential and the concentrations decay slowly towards their initial values. The decay time constant of sodium for the chosen simulation parameters can be estimated as $\tau _{Na^+} = V_{Head} l_{Neck} / R_{Neck} / D_{Na^+} = 19.6~ms$ (Tønnesen & Nägerl, 2016). $R_{Neck}$ is the total resistance of the spine neck. In the simulation a decay time constant of 19.2 *ms* is found, consistent with the predicted value.

**Fig. 3:**
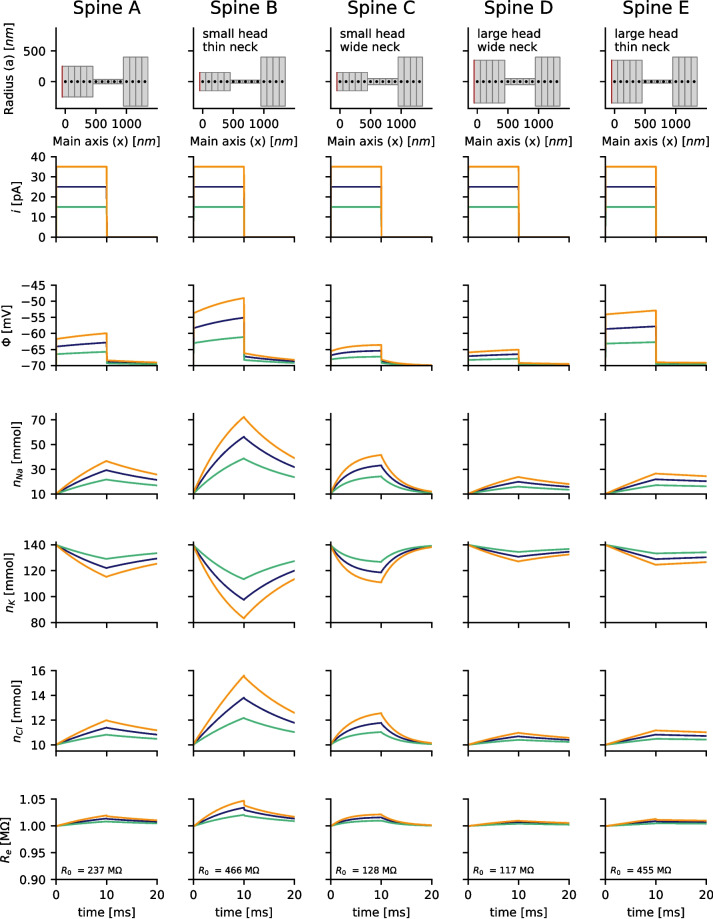
Changes in the membrane voltage $\Phi$ and the ion concentrations of sodium $n_{Na}$, potassium $n_{K}$ and chloride $n_{Cl}$ related to the current injections (step current from $t=0$ to $t=10ms$) strongly depend on the spine morphology, which is highly variable for pyramidal cells. Different currents *i* and diameters of idealized neck and head configurations are compared. Spine A corresponds to the spine shown in Fig. 1. In smaller spine heads the concentration changes are stronger than in larger spine heads. A thinner spine neck also increases the depolarization and concentration changes. The total electric resistance of the spine $R_e$ for drift currents increases as a result of the concentration changes. $R_0$ denotes the electric resistance at time $t=0 ms$. The electric potential and the ion concentrations were measured in the leftmost segment of the spine head

Experimental measurements usually show a high variance of the EPSP peak voltages in spines (Cornejo et al., 2022), and other studies also measure higher voltages or currents (Acker et al., 2016; Kwon et al., 2017) as assumed for the simulation of Fig. 1. This is in agreement with spines existing in various shape and size. The spine morphology gets usually quantified by the head volume, the neck width and the neck length. The potential and the ion concentrations for different shape parameters and different strength of input current are simulated (Fig. 3). As expected the neck diameter is the main determinant of the neck resistance (if the length is identical). The depolarization of the spine head is stronger in spines with thin necks. Moreover, in smaller spine heads the concentrations changes are stronger, than in large spines (compare spine B with spine E and spine C with spine D). Especially in spines with small head volumes and thin necks (Fig. 3, spine B) the the ion concentrations change considerably. The sodium concentration increased from 10 mM to more than 70 mM, while the potassium concentration dropped accordingly.

### The accurate choice of parameters is crucial for simulations of dendritic spines

As a result of the concentration changes, a previous modelling study based on PNP-equations predicted that the intracellular resistance decreases during synaptic input (Lagache et al., 2019). However, an analysis of the total resistance (see Eq. (22)) along the spine during current injection, shows that the resistance slightly increases instead in this study (Fig. 3, last row). A major difference between the two studies is that here sodium, potassium and chloride are considered as different ion-species at physiological ion concentrations and with different diffusion constants while (Lagache et al., 2019) distinguish only between positive and negative ions and assume that both ion types have the same concentration and diffusivity. Therefore, the question arises: How do the simulation results depend on the particular choice of parameters?

**Fig. 4:**
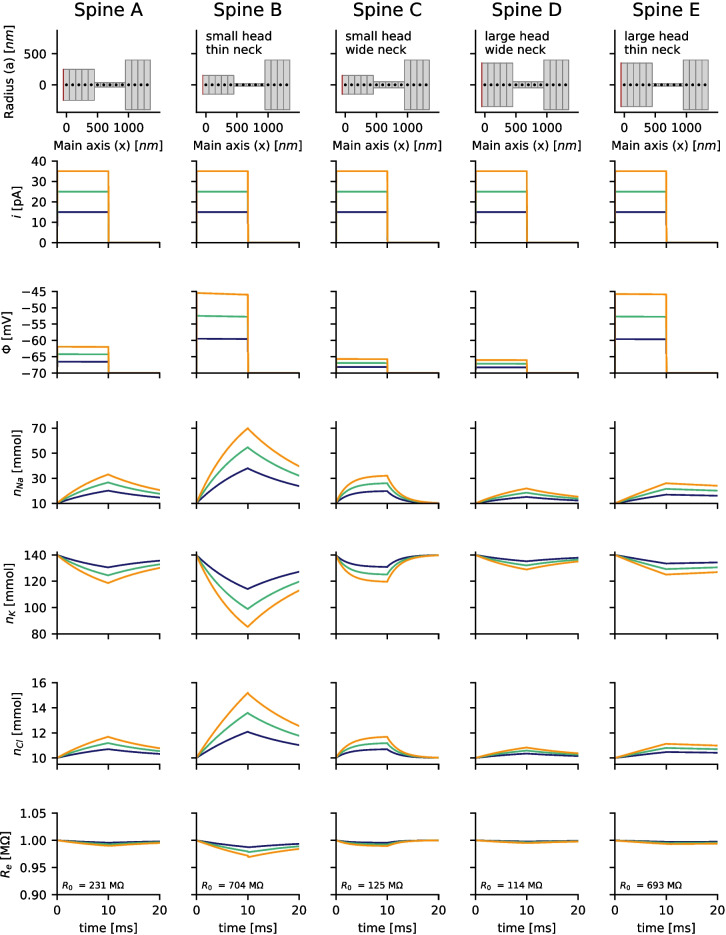
The simulations presented in Fig. 3 were repeated with a higher diffusivity of sodium, where $D_{Na^+}=D_{K^+}=D_{Cl^-}=1.00 \cdot 10^{-9} \frac{m^2}{s}$. Changes in the membrane voltage $\Phi$ and the ion concentrations of sodium $n_{Na}$, potassium $n_{K}$, and chloride $n_{Cl}$ are illustrated for different injected currents *i* (step current from $t=0$ to $t=10,ms$). Unlike in Fig. 3, the membrane depolarization can now be computed using Ohm’s Law as the product of the total resistance for drift currents $R_e$ and the strength of the injected current *i*. However, compared to Fig. 3, the total resistance $R_e$ now decreases. $R_0$ denotes the electric resistance at time $t=0,ms$. The electric potential and the ion concentrations were measured in the leftmost segment of the spine head

**Fig. 5:**
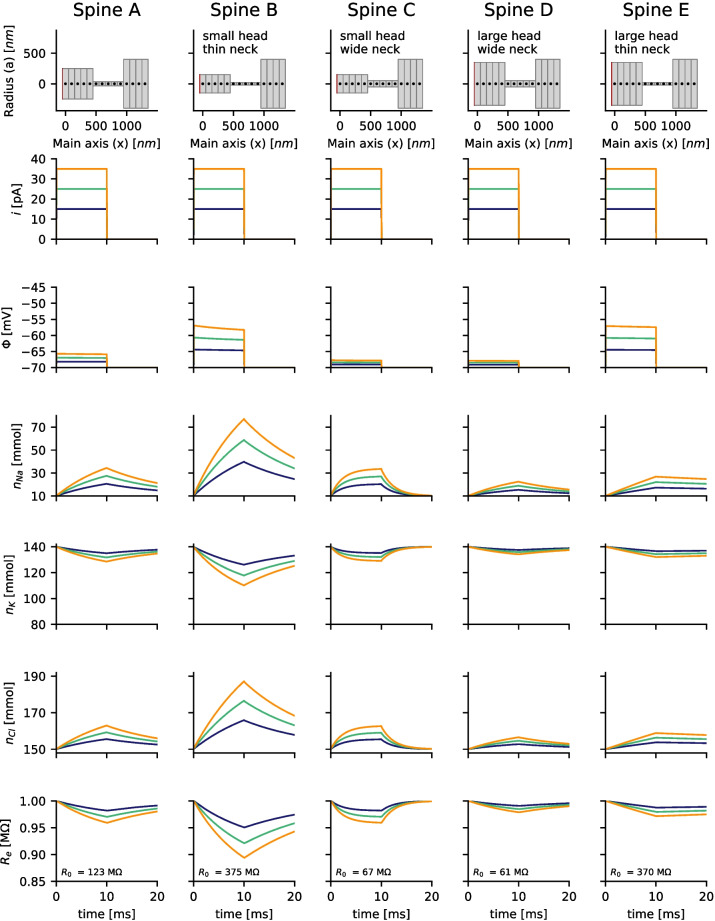
The simulations presented in Figs. 3 and 4 are repeated. The diffusion constants of sodium, potassium, and chloride remain identical as in Fig. 3, $D_{Na^+}=D_{K^+}=D_{Cl^-}=1.00 \cdot 10^{-9} \frac{m^2}{s}$. The resting concentration of chloride is set to 150 mM, and the negative background charge was removed. The number of free anions and free cations is now identical. Changes in the membrane voltage $\Phi$ and the ion concentrations of sodium $n_{Na}$, potassium $n_{K}$, and chloride $n_{Cl}$ are illustrated for different injected currents *i* (step current from $t=0$ to $t=10,ms$). The discrepancies from the simulation results of Fig. 3 become even more pronounced. The total resistance for drift currents $R_e$ can be significantly decreased during current injection. $R_0$ denotes the electric resistance at time $t=0,ms$

To better understand the discrepancies in the results of (Lagache et al., 2019) and this study, the simulations presented in Figure 3 are repeated, but with altered parameters. As in (Lagache et al., 2019), the diffusion constant of sodium is set to be equal that of potassium and chloride. Interestingly the cumulative resistance is now decreasing during current injection (Fig. 4). Next, the concentration of chloride is increased to 150 mM, while reducing the number of immobile background charges. Positive and negative ions now have the same concentration and diffusivity. In this case the decrease of the resistance became even more prominent (Fig. 5) and comparable to (Lagache et al., 2019). To explain this, two opposite effects are important:

First: Sodium has a 35% reduced diffusivity compared to potassium. In cases where sodium is accumulated and potassium is depleted by the same amount, the average diffusivity of the ions drops (averaged across all ion species). As the electrical resistance is inversely proportional to the diffusion constants of the ions (see Eq. (3)), the total resistance increases.

Second, to compensate for injected sodium ions, potassium cations leave the spine, but chloride anions also enter the spine. The system remains approximately electroneutral, but, in total, more charges enter the spine (sodium and chloride) than leave it (potassium). As the number of ions in the spine increases, the conductivity of the intracellular electrolyte increases, and the resistance $R_e$ for drift currents drops. This effect becomes stronger when more chloride is available in the system.

In the original setup of this study (Fig. 3), the first effect dominates over the second effect, and the total resistance has to increase.

In the second setup (Fig. 4), where all diffusion constants are equal, the first effect vanishes. The second effect leads to a decrease in the spine resistance $R_e$.

In the third case, where more chloride ions are available in the system (Fig. 5), roughly half of the influx of sodium through the synapse is compensated by an influx of chloride through the spine neck. The second effect becomes even stronger, and the accumulation of ions extends into the spine neck. This can cause a significant drop in neck resistance (e.g., Fig. 5 Spine B).

In summary, it is essential to include physiological ion concentrations and correct diffusion constants to accurately study the spine neck resistance and the electrical function of dendritic spines in numerical simulations.

### Diffusion currents boost the spine depolarisation

As shown before the spine head’s membrane potential is continuously increasing during current injection (Fig. 3, third row). This effect cannot be explained by the small changes of the intracellular resistance (Fig. 3, last row). To understand how the head’s membrane potential is changing, next, the electrical currents are analyzed in detail. These currents can be separated into currents caused by the electric field and currents caused by ion-concentration gradients.

**Fig. 6:**
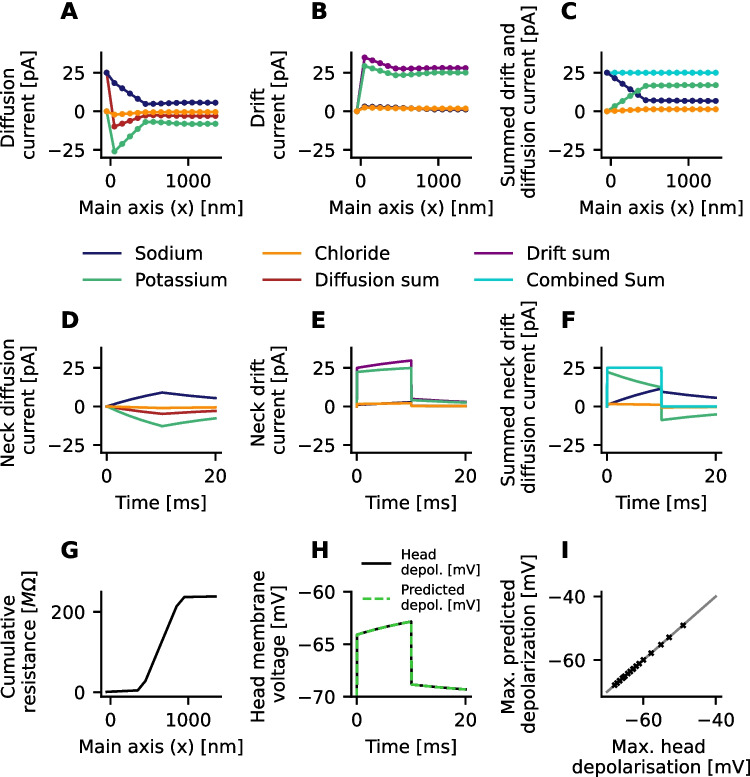
The electric currents of the simulation shown in Fig. 1 are broken down. **A** Electric currents evoked by the diffusion of ions between the lattice points after 10 ms of current injection (25 pA). **B** Electric currents evoked by the electric field (drift current) after 10 ms of current injection. **C** Sum of the currents shown in **A** and **B**. The total electric current (sum of drift and diffusion currents) exactly matches the injected current everywhere in the spine. An increased electric field therefore compensates the diffusion of electric charges. **D** Diffusion currents through spine neck. **E** Drift currents through spine neck. **F** Summed current through spine neck exactly matches the injected current. **G** The cumulative resistance along the spatial lattice after 10 ms. Almost all of the resistance is caused by the spine neck. H) The product of the total current evoked by the electric field (purple line in B) and the total resistance (max. value of cumulative resistance as shown in **G **theoretically predict the measured membrane voltage in the spine head. Diffusion currents, therefore increase the membrane voltage in spines. **I** Identical to the case shown in **H** the theoretically predicted depolarization and the measured depolarization after 10 ms match for all simulations shown in Fig. 3

As shown in Figure 1E-G and Figure 2 the ion concentrations are considerably changing in the spine head during current injection. Along with the increasing concentration gradients, this increases the diffusion of ions and leads to an measurable electrical current (diffusion current). Indeed, after 10 ms of current injection considerable diffusion currents are found (Fig. 6A). Currents in the head are larger because of the larger radius a. Sodium and chloride ions are diffusing out and potassium ions are diffusing into the spine head. In total, the sum of all diffusion currents is negative.

Next (Fig. 6B), currents along the spine’s main axis induced by the electric field (drift current) were analyzed. All drift currents are positive, so that, on average, the electric field forces sodium and potassium ions to leave the spine head and chloride ions to enter the spine head. Most of the drift current is caused by potassium ions, which have the highest concentration of all ions in the intracellular space. Interestingly, the summed electric current is higher than the injected current of 25 pA. But when the sum of all currents is computed (Fig. 6C), it can be seen that diffusion and drift currents exactly add up to 25 *pA*, everywhere in the spine, so that they are equal to the injected current. In summary, as the ion concentrations are changing, this leads to diffusion currents. To maintain a balance between positive and negative charges, the electrical currents have to compensate the diffusion currents.

Subsequently, the different currents through the spine neck over time are studied. As the concentration gradients between the head and dendrite increase, the diffusion currents get stronger but have a negative value in total (Fig. 6D). The diffusion currents persist after the end of the current injections at 10 ms. The drift currents through the spine neck (Fig. 6E) increase over time. A small drift current continues after the end of the current input. The total neck current always matches the injected current exactly (Fig. 6F).

Finally, the cumulative electrical resistance of the simulated spine is analyzed (Fig. 6G). Most of the total resistance is caused by the spine neck. Above, it was found that the total resistance $R_e$ only slightly changes over time (Fig. 3, last row). Based on the injected current (Fig. 3, second row) and Ohm’s law, this cannot account for the changes in the head depolarization (Fig. 3, third row). However, when the voltage drop across the simulated spine is estimated from the total electrical resistance $R_e$ (Eq. (22)) together with the drift current $I_e$ (purple line in Fig. 6E), $\Phi _{\text {est}} = \sum _i I_e(x_i) r_e(x_i) \Delta x / A(x_i)$ instead of the injected synaptic current (Fig. 6F, cyan line), the spine head voltage can be accurately predicted (Fig. 6H). To verify this for other spines, the maximum value over time of the predicted head voltage is compared with the maximum value of the measured head membrane potential (Fig. 6I). Both values agree for all spines and input currents shown in Figure 3.

**Fig. 7:**
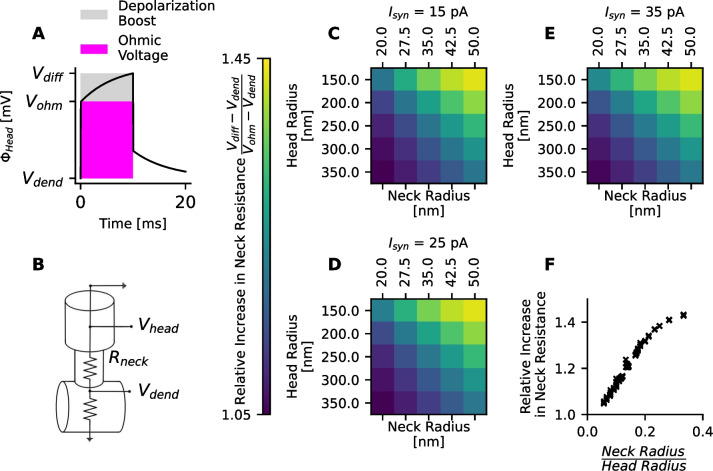
Diffusion currents increase the experimentally measured neck resistance. **A** A constant current $I_{syn}$ is injected for 10 ms. Several microseconds after the onset of the current injection, the head’s membrane capacitor is depolarized to a value $V_{\text {ohm}}$. During the current injection, the influx of sodium through the synaptic end leads to a build-up of concentration gradients across the neck. The resulting diffusion currents boost the membrane potential in the head, $\Phi _{\text {head}}$, up to a value $V_{\text {diff}}$ after 10 ms. **B** Experimentally, the spine neck resistance can be inferred from measurements of the electric current and the membrane depolarization in the head and in the dendritic shaft. In the simulations, the electric current is constant and resembles the injected current. The estimated neck resistance increases as the depolarization of the spine head, $\Phi _{\text {head}}$, increases compared to the depolarization of the dendritic shaft. **C**-**E** To test different spine morphologies, the radii of the head and neck segments are varied. The relative increase in the estimated spine neck resistance after 10 ms of current injection is measured for different strengths of the injected current: 15 pA in (**C**), 25 pA in (**D**), and 35 pA in (**E**). **F** Diffusion currents can increase the estimate of the spine neck resistance by up to 45%. The relative increase depends on the ratio between neck radius and head radius, but not on the synaptic current

In summary, the ion-concentration gradients lead to a net diffusion current into the spine head. These diffusion currents are compensated by an increase of the electrical drift currents, which in turn leads to an increase of the spine head membrane potential (Fig. 7A).

### Diffusion currents increase measured values of the spine neck resistance

Experimentally, the spine neck resistance is often estimated based on a voltage divider (Eq. (23) and Fig. 7B). For this purpose, the synaptic current and the depolarization in the spine head and the dendritic shaft are measured. Then, the spine neck resistance is calculated based on Ohm’s law. However, above it was shown that during synaptic input, concentration gradients across the spine neck can build up. The resulting diffusion currents increase the spine head’s membrane potential, while the total electric current through the spine neck remains constant (Fig. 6F). Consequently, as soon as there are diffusion currents, the linear relationship between synaptic current and spine head membrane depolarization is lost, and the spine neck resistance does not behave like an Ohmic resistor anymore. Therefore, the question arises, whether diffusion currents will affect measurements of the spine neck resistance.

To estimate the effect of the diffusion currents on measurements of the spine neck resistance, a constant sodium current is injected for 10 ms. Several microseconds after the onset of current injection, the membrane is charged to a value $V_{ohm}$ (Fig. 7A). In this initial time of capacitor charging, the ion concentrations remain unchanged, and the spine neck resistance is an Ohmic resistor. In the following 10 ms of current injection, concentration gradients build up and boost the depolarization of the spine head to a value $V_{diff}$ (Fig. 7A). As the injected current is constant, the inferred spine neck resistance is directly proportional to the depolarization difference between the head and dendrite. The relative increase *B* of the measured neck resistance $R_N$ after 10 ms is, therefore, $B = \frac{ V_{diff}-V_{dend}}{V_{ohm}-V_{dend}}$. This relative increase was measured for three different strengths of the injected current, 15 pA in (7C), 25 pA in (7D), and 35 pA in (7E). To test different spine volumes and neck resistances, the radii of the head and neck segments are varied. In all three cases, a relative increase in the spine resistance was up to 45% (7F). Even though the absolute depolarization depends on the injected current, the relative increase only depended on the ratio between the radii of the head segments and the neck segments.

### Interactions between synaptic current injection and dendritic depolarization

The coincidence of pre- and postsynaptic action potential can trigger synaptic plasticity in dendritic spines (Sweatt, 2016). The direction of the changes in synaptic strength are determined by the temporal order of pre- and postsynaptic activity. The magnitude is positively correlated with the peak elevation of the intracellular $Ca^{2+}$ concentration (Nevian & Sakmann, 2006).

The primary pathway for $Ca^{2+}$ influx from the extracellular space into dendritic spines is via NMDA-type glutamate receptors (NMDARs). The channel conductance of NMDARs is, via the $Mg^{2+}$ block, highly voltage dependent (Bloodgood et al., 2009). But the membrane voltage in spine heads gets boosted, as found before, by ion diffusion through the spine neck after current injection. Therefore the question arises how the strength, duration and the timing of synaptic input could affect the NMDAR-channel mediated current during sequences of pre- and postsynaptic action potentials.

To examine if diffusion currents can influence NMDAR-mediated calcium current, the voltage-dependent activity of NMDAR channels is analyzed. Given the low levels of calcium concentration and current in comparison to sodium, potassium, and chloride, the contribution of calcium to the membrane potential is disregarded (Li, 2023). All synaptic current is again mediated by an influx of sodium.

**Fig. 8:**
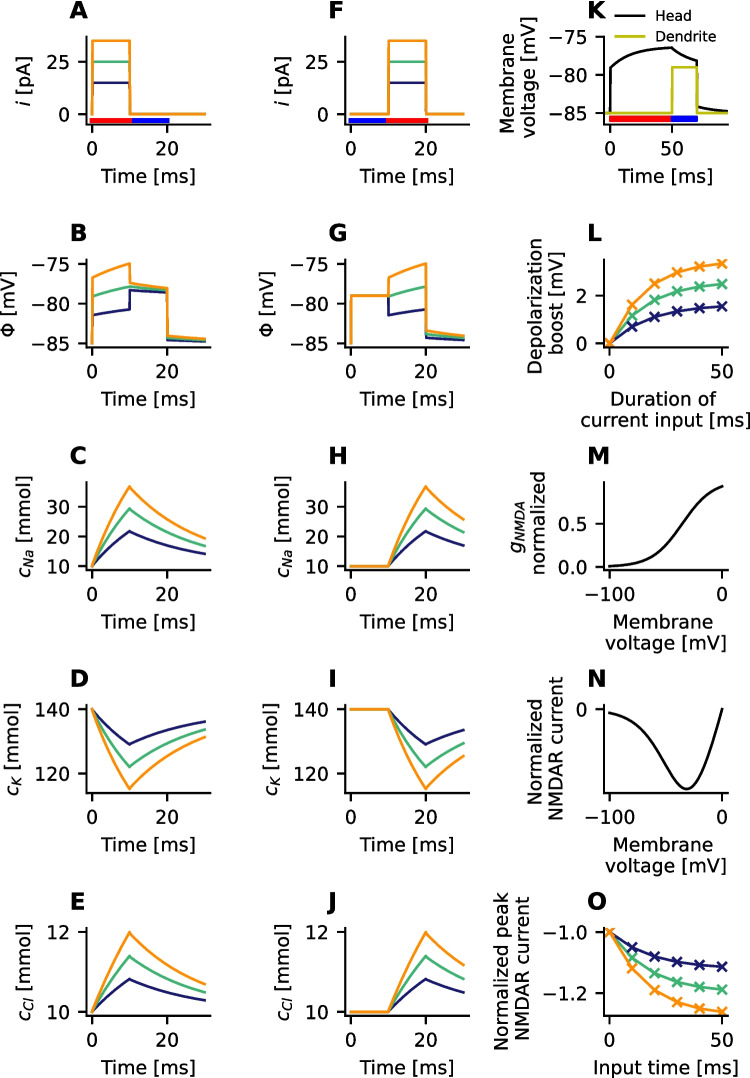
**A**, **F**, **K** The simulation protocol consists of a subsequent injection of current and a depolarization by 6 mV at the dendritic end. The phase of synaptic current injection is indicated by a red bar, whereas the phase of dendritic depolarization is indicated by a blue bar. The depolarization of the dendrite mimics a dendritic depolarization caused by a back-propagating action potential (bAP). **B**-**E** The electric potential $\Phi$ and the ion concentrations of sodium $n_{Na}$, potassium $n_K$, and chloride $n_{Cl}$ in the spine head are observed when a current *i* is injected for 10 ms, followed by the depolarization of the dendrite by 6 mV for the next 10 ms. **G**-**J** The electric potential and the ion concentrations in the spine head when the dendrite is first depolarized by 6 mV for 10 ms, and then a current is injected for the next 10 ms. **K** A variable duration (red bar) of the injected current is used to model variable presynaptic activity. Membrane potential and NMDAR-current are analyzed in the subsequent phase of dendritic depolarization (blue bar). **L** Increasing the duration and the strength of the current injection boost the spine head’s depolarization. The shown voltage values will add to the 6 mV depolarization of the head caused by the dendritic depolarization. **M** Peak-normalized conductivity of NMDARs grows as a function of voltage. **N** Normalized NMDAR current as a function of holding potential. **O** Peak NMDAR current during the phase of dendritic depolarization. The values are scaled by the peak NMDAR current, when there is no depolarization boost. The increased membrane voltage amplifies the NMDAR-current. **M**-**O** The model of the channel conductance $g_{NMDA}$ and the NMDAR current are reproduced from (Chiu & Carter, 2022), see Methods. The electric potential and the ion concentrations were measured in the leftmost segment of the spine head (Fig. 1B)

To test whether the spine depolarization depends on the chronological order of pre- and postsynaptic action potential, two cases are compared. In the first case a current (15 - 35 pA) is injected for 10 ms into the spine head (parameters chosen as in Fig. 8A). Subsequently the dendritic end gets depolarized by 6 mV for 10 ms (Fig. 8B). In the second case the dendritic end is depolarized first (Fig. 8G), followed by a current injection into the spine head (Fig. 8F). In the first case the ion concentrations are altered after 10 ms of current injection (Fig. 8C–E). The diffusion currents persist into the phase of dendritic depolarization and raise the membrane voltage of the spine head above the dendritic depolarization of 6 mV (Fig. 8B). Differently in the second case; the depolarization of the dendrite propagates into the spine head without attenuation (Fig. 8G). The ion concentrations are unaffected and remain constant inside the spine head during the phase of dendritic depolarization (Fig. 8H–J). There are no lasting effects for the following time of current injection.

Therefore, this study focuses on sequences where a synaptic input is followed by a postsynaptic action potential. The boost of the spine head’s membrane voltage during postsynaptic activity depends on the synaptic input. To quantify this dependency a current of variable strength and duration is injected (red bar in Fig. 8K indicates duration of current injection). Right after the current injection, the dendrite gets depolarized by 6 mV for 10 ms again (blue bar in Fig. 8K). It can be seen that when the duration of current injection is increased from 10 ms to 50 ms the depolarization boost increases from 0.70 mV to 1.55 mV for 15 pA current strength and from 1.62 mV up 3.34 to mV for 35 pA current (Fig. 8L). Based on 6 mV depolarization in the dendrite the heads membrane voltage gets amplified by up to 55.6 % in the tested cases.

The NMDAR channel conductance increases as a function of the membrane voltage (Fig. 8M). This leads to a higher NMDAR current in the relevant voltage range (Fig. 8N). Therefore the peak NMDAR current is computed as function of the input strength and duration. It is found that current through NMDARs can be boosted by roughly up to 25% for realistic spine parameters.

In summary, repeated synaptic input results in a boost of the spine head’s membrane potential that persists for several milliseconds. This increase is substantial enough to enhance NMDAR currents during an immediately subsequent back-propagating action potential (bAP). This effect has the potential to amplify calcium influx if a back-propagating action potential (bAP) follows synaptic input.

## Discussion

Dendritic spines have a very small volume. As a consequence, physiological electric currents can alter the ionic composition of the spine’s intracellular space. The resulting concentration gradients lead to additional electric currents caused by the diffusion of ions. These currents are not captured by the cable equation (Rall, 1977), as it only considers the electric field as driving force of ion movement. The PNP-equations on the other hand are capable of accurately estimating the ion-movement in spines based on electrodiffusion. But due to the high complexity of this equation system, they are not suited to find solutions in larger systems with multiple ion species.

This study demonstrates the derivation of an extension to cable theory that incorporates diffusion currents, utilizing an analogy between electric potential and chemical potential. The resulting set of equations can be effectively solved using a straightforward explicit algorithm based on a finite difference scheme. The utilization of electrodiffusion models to simulate electric signals in spines has been explored in prior works (Breit & Queisser, 2021; Lagache et al., 2019). The concepts employed in this derivation have previously been applied to derive fractional cable equations (Henry et al., 2008). Similar equations have found application in modeling calcium signals in dendrites Li (2023). This paper extends several of these concepts to spines and introduces a finite difference scheme, facilitating rapid implementation and ensuring the reliable modeling of electrodiffusion in spines.

Throughout this study, ionic currents in dendritic spines during current injection are simulated. The spine parameters are chosen in agreement with experimental findings (Cornejo et al., 2022). Based on computer simulations, a previously unrecognized mechanism that boosts spine depolarization during synaptic input is identified here. During the simulation, the ion concentrations in dendritic spines are considerably changing. The sodium concentration increases by almost 200% after 10*ms* of a 25*pA* input current, while potassium drops by the same amount. The concentration changes are in agreement with previous simulation studies based on PNP-equations (Lagache et al., 2019; Qian & Sejnowski, 1989). Despite concentration changes, the spine maintains electroneutrality automatically, and the changes in sodium and potassium concentration in the spine head approximately compensate. However, the accumulation of sodium and a depletion of potassium in the spine head lead to measurable diffusion current through the spine neck. Due to the distinct diffusion constants of sodium and potassium, the concentration changes lead to a measurable net diffusion current. The summed current evoked by diffusion flows in the opposite direction compared to the current evoked by the electric field. To further maintain the balance between positive and negative charges in the spine head, the electric field increases and compensates for the net diffusion of ions. As a result, the depolarization of the spine head rises by up to 45%, depending on the spine morphology.

To better understand this phenomenon, one can relate it to the Goldman equation. Here, the membrane potential evoked by sodium, potassium, and chloride depends on the exact ionic concentrations on both sides of the membrane and the different permeabilities of the three ion species inside the membrane. The permeability in the Goldman equation can be compared to the neck resistance for each ion, and the intracellular and extracellular ion concentrations correspond to head and dendrite ion concentrations. An important difference, however, is that the effective current across the membrane is zero, and the system is stationary in the Goldman equation, while here the system is non-stationary, and a current flows through the spine neck.

To accurately study this mechanism, the correct choice of diffusion constants and physiological ionic concentrations is critical. If parameters are poorly chosen, this affects the resulting ion concentrations and diffusion currents, and the findings can even be reversed. This is evident in the spine neck resistance. At physiological ion concentrations and realistic diffusion parameters, the spine neck resistance slightly increases. But in a system with equal diffusion constants, an increased concentration of chloride causes the spine neck resistance to drop instead. This can explain differences with existing literature (Lagache et al., 2019).

The described mechanism has important implications for measurements of the spine neck resistance. The build-up of diffusion current increasingly boosts the spine head’s depolarization while the injected current remains constant. Therefore, the linear relationship between depolarization and current is lost, and Ohm’s law is violated for the spine neck resistance. However, if an Ohmic resistor is assumed for measurements of the spine neck resistance, this effect can lead to a significant overestimation of the neck resistance.

The study explores interactions between synaptic input and postsynaptic activity. For this purpose, a current was injected through the synapse, followed by a subsequent depolarization of the dendritic end. The current injection leads to changes in ionic concentrations that persist during subsequent dendritic depolarization. The magnitude of concentration changes depends on the strength and duration of the synaptic input. The resulting diffusion currents then amplify the depolarization in the spine head, augmenting the depolarization originating from the dendrite. The amplified membrane depolarization in the head increases the conductance of NMDAR channels and can thereby elevate the calcium influx into the spine. In average pyramidal cell spines, this effect can increase peak calcium influx by up to 25% after strong current inputs, as expected during repeated activation of the synapse, e.g., during a presynaptic spike train. This effect depends on the spine’s morphology and is especially important for small spines with long and thin necks. These findings indicate that diffusion currents can be beneficial for synaptic plasticity. Synaptic plasticity can be triggered by a pairing of pre- and postsynaptic action potentials (Lamprecht & LeDoux, 2004). Thereby, the temporal order is important to set the direction of changes in synaptic strength. Postsynaptic activity that follows synaptic input within a short time can lead to long-term potentiation of the synapse. The peak elevation of $Ca^{2+}$ concentration controls the magnitude of the changes (Nevian & Sakmann, 2006).

The equations underlying the simulations here can be considered as a generalization of the cable equation. By setting the ion concentrations to a constant value, one recovers the well-known form of the cable equation. By discretizing the cable along the main axis into compartments of finite size, one can build multi-compartment models to study large dendritic trees (Eberhardt et al., 2019). The same concept was applied to the equations presented here. This can be utilized to extend compartmental models and to accurately estimate synaptic currents, including the diffusion of ions. Finally, the inclusion of larger molecules into the model, such as dye molecules used for photo-stimulation, might also help to correctly interpret experimental results from imaging spines.

## Data Availability

Not applicable.

## Footnotes

1 $N=V\cdot N_A\cdot c$, where *N* is the number of ions, $N_A$ the Avogadro constant, and *c* the concentration and V the volume.

2 $N = i_{syn} \cdot \Delta t / e$, where N is the number of ions, $i_{syn}$ the average current strength, *e* the elementary charge and $\Delta t$ the duration.

## Code availability

All the work was performed by the corresponding author.

## Declarations

### Employment

During the time of the work on this manuscript, the author was and is employed at the Ludwig-Maximilians Universität München.
